# Expression of Na/K-ATPase subunits in the human cochlea: a confocal and super-resolution microscopy study with special reference to auditory nerve excitation and cochlear implantation

**DOI:** 10.1080/03009734.2019.1653408

**Published:** 2019-08-28

**Authors:** Wei Liu, Maria Luque, Rudolf Glueckert, Niklas Danckwardt-Lillieström, Charlotta Kämpfe Nordström, Anneliese Schrott-Fischer, Helge Rask-Andersen

**Affiliations:** a Department of Surgical Sciences, Section of Otolaryngology, Uppsala University Hospital, Uppsala, Sweden;; b Department of Otolaryngology, Medical University of Innsbruck, Innsbruck, Austria;; c Department of Surgical Sciences, Head and Neck Surgery, Section of Otolaryngology, Uppsala University Hospital, Uppsala, Sweden

**Keywords:** Auditory nerve, cochlea, human, Na/K-ATPase, structured illumination microscopy

## Abstract

**Background:** For the first time the expression of the ion transport protein sodium/potassium-ATPase and its isoforms was analyzed in the human cochlea using light- and confocal microscopy as well as super-resolution structured illumination microscopy. It may increase our understanding of its role in the propagation and processing of action potentials in the human auditory nerve and how electric nerve responses are elicited from auditory prostheses.

**Material and methods:** Archival human cochlear sections were obtained from trans-cochlear surgeries. Antibodies against the Na/K-ATPase β1 isoform together with α1 and α3 were used for immunohistochemistry. An algorithm was applied to assess the expression in various domains.

**Results:** Na/K ATPase β1 subunit was expressed, mostly combined with the α1 isoform. Neurons expressed the β1 subunit combined with α3, while satellite glial cells expressed the α1 isoform without recognized association with β1. Types I and II spiral ganglion neurons and efferent fibers expressed the Na/K-ATPase α3 subunit. Inner hair cells, nerve fibers underneath, and efferent and afferent fibers in the organ of Corti also expressed α1. The highest activity of Na/K-ATPase β1 was at the inner hair cell/nerve junction and spiral prominence.

**Conclusion:** The human auditory nerve displays distinct morphologic features represented in its molecular expression. It was found that electric signals generated via hair cells may not go uninterrupted across the spiral ganglion, but are locally processed. This may be related to particular filtering properties in the human acoustic pathway.

## Introduction

The human inner ear is difficult to study due to its exceptional fragility and the surrounding hard bone, and there are few descriptions of its molecular physiognomies. Further information is necessary to understand how acoustic information from inner hair cells (IHCs) is relayed to the acoustic nerve and central nervous system (CNS). Also, it is not known how and where extra-cellular electric stimulation from cochlear auditory prostheses, an outstanding achievement in modern medicine, elicits nerve responses to the CNS. Important information has been gained from animal models, but translational research has been hampered by noticeable structural differences reflecting functional species diversities.

Na/K-ATPase is involved in several specialized functions in the ear. Several studies have been performed in animals but only a few in the human cochlea (1,2). Recently, we used super-resolution structured illumination microscopy (SR-SIM) to analyze the molecular structure of the human ion machinery in the lateral cochlear wall (2). This unique tissue generates the endo-cochlear potential essential for mechanic-electric transduction (3,4). Its function depends on selective ion transport channels, high energy-consuming Na/K-ATPase pumps, and unique intercellular barriers.

Na/K-ATPase contains four catalytic α isoforms, three β, and seven FXYD subunits (5–8). The α1 subunit is expressed in all cells, and the α1β1 heterodimer is found in kidney tubules (9). The β form is believed to be involved in the folding and transport of the synthesized catalytic α subunits (10). Fast nerve excitation depends on Na/K-ATPase for restoration of membrane potential following depolarization, but also the regulation of cell volume and signal transduction (11,12). The α3 isoform is typically expressed in neurons to transfer ions across the cell membrane of hyperpolarized cells. The important role of the neuron-specific α3 subunit in the ear was shown by the severe effects of mutations encoding the α3 subunit (13) and its sensitivity to ouabain (14–16). We used SR-SIM and confocal microscopy (CM) to display Na/K-ATPase isoforms in the human cochlea, including the auditory nerve. This technique allows immune investigation of proteins with high resolution beyond the diffraction limits (17–20). We analyzed the relative amount of β1 isoform expression in various parts of the cochlea using false color imaging software. The primary goal was to shed more light on its role in the processing of action potentials in the human auditory nerve. It is also undetermined how and where action potentials are generated in cochlear implant recipients.

## Material and methods

### Ethical statements

Well-preserved human cochlear tissue was obtained with excellent antigen preservation, shown in our previous studies (20). The study of human cochleae was approved by the local ethics committee (Etikprövningsnämnden Uppsala, no. 99398, 22/9 1999, cont., 2003, no. C254/4; no. C45/7 2007, Dnr. 2013/190), and patient consent was obtained. The study adhered to the rules of the Declaration of Helsinki. The surgical specimens (cochleae) were from patients suffering from life-threatening posterior cranial fossa meningioma when the cochlea had to be destroyed due to severe brain stem compression.

### Collecting and processing human cochlear tissue

To obtain freshly fixed tissue from the human inner ear is challenging. The inner ear is surrounded by the hardest bone in the body, and tissues undergo rapid post-mortem degeneration. This rarely available surgical material offers unique possibilities due to direct fixation, but suffers from the limited study of its variability and control procedures. Fresh samples of tissue were collected during trans-cochlear surgery for meningioma that reached the clivus. No personal patient data were retrieved or stored, according to the Biobank law. Six cochleae were dissected out using diamond drills of various sizes. Single-cell RNA analyses and gene-profiling were performed, but these were unsuccessful due to the long decalcification time. SR-SIM and antibody co-expression analyses of human spiral ganglion cells were carried out and combined with transmission and scanning electron microscopy (TEM and SEM). Cochlear tissue was directly fixed in glutaraldehyde for electron microscopy. Western blot and *in situ* hybridization were not performed due to the limited amount of tissue. TEM analyses were made in two human specimens that were analyzed at inner ear research laboratories in both Uppsala and Innsbruck (21). Innsbruck University also analyzed donated human temporal bones obtained at autopsy. Two cochleae were also used that were previously processed and morphologically analyzed (22). Both were from middle-aged females with normal hearing. Cochleae were decalcified in 0.1 M Na-ethylene-diamine-tetra-acetic acid (EDTA), pH 7.4, for 6 weeks.

### Immunohistochemistry

#### Surgical tissue—SR-SIM and confocal microscopy (CM)

The study is based on the same material of archival sections of the human cochlea and immunohistochemistry procedures described in previous publications (2,23). Tissue was directly fixed in a solution of 4% buffered paraformaldehyde. The cochleae were decalcified in 10% EDTA solution at pH 7.2 and embedded in Tissue-Tek (OCT Polysciences, Inc., Warrington, PA, USA), rapidly frozen, and sectioned at 8–10 μm using a cryostat microtome. The frozen sections were stored below −70 °C before immunohistochemistry. The sections used for antibody control were incubated with 2% bovine serum albumin (BSA), omitting the primary antibodies. Primary and secondary antibody controls and labeling controls were used to exclude endogenous labeling and reaction products (24). Control sections were incubated with 2% BSA, omitting the primary antibodies. The control experiment revealed no visible staining in any structure of the cochleae. The antibodies used for IHCs are shown in Table 1. Due to the close location of spiral ganglion neurons and the glial cells, identifying the distribution of Na/K-ATPase subtypes in the nerve versus glial cell depends on a microscope with high resolution and multi-channels. SR-SIM was performed at Uppsala SciLife national facilities (http://www.scilifelab.se/#). The resolution of the SIM system (BioVis, Uppsala University) used in the present study was measured with sub-resolution fluorescent beads (40 nm) (Zeiss, Oberkochen, Germany) in the green channel (BP 495–550 + LP750) (25). SR-SIM gave a lateral precision of 80 nm. To analyze the intensity variation of fluorescence of the protein, a ‘false color algorithm’ was used with a length scale of 5 μm or 10 μm representing different intensities of fluorescence (16 or 32 colors). TEM was performed at the Ear, Nose and Throat Department at Uppsala University Hospital using a JEOL 100SX microscope (Tokyo, Japan). Scanning electron microscopy was done at the Department of Cell Biology in Innsbruck using a Zeiss DSM982 Gemini field emission scanning electron microscope at 5 kV. Maximum resolution was estimated to 2 nm. Coating was performed with gold-palladium to a nominal depth of 10–12 nm.

**Table 1.** Antibodies used in the present investigation.

Protein	ab	Dilution	Species	Catalog no.	Provider
Na/K-ATPase (α1)	monoclonal	1:50	mouse	NB300-146	Novus
Na/K-ATPase (α1)	monoclonal	1:500	mouse	C464.6	Merck-Millipore
Na/K-ATPase (α3)	monoclonal	1:100	mouse	NB300-540	Novus
Na/K-ATPase (α3)	polyclonal	1:800	goat	sc-16052	Santa Cruz Biotechnology
Na/K-ATPase (β1)	monoclonal	1:100	mouse	Ma3-930	Abcam
Nav1.6	monoclonal	2:1	mouse	73-026	Department of Neurobiology, Physiology and Behavior, UC Davis, Davis CA 95616-8519
Cx30	polyclonal	1:100	rabbit	71-2200	Invitrogen
MBP	polyclonal	1:100	rabbit	AB980	Merck-Millipore

#### Human temporal bones (TBs)

Human bodies were donated to the Division of Clinical and Functional Anatomy of the Innsbruck Medical University by individuals who had given their informed consent prior to death for the use of their bodies for scientific and educational purposes (26,27). All temporal bones were anonymized. There was no evidence for any malformation in any human temporal bone. The post-mortem delay until fixation in 4% buffered formaldehyde varied from a few hours up to 13.5 hours. Sex and ages of individuals were not reported due to anonymization rules. Round and oval windows were opened with a preparation needle and fixative gently perfused into perilymph. TBs were left in fixative for 48 hours on a shaker at 4 °C followed by several washes in phosphate buffer. Excessive tissue and bone were removed with bone forceps followed by drilling the TB to the otic capsule. The thinned-out inner ear was decalcified for 17 to 20 hours in EDTA 20% (Merck, Darmstadt, Germany) at 37 °C and fluids circulated with a magnetic stirrer. All tissues were cryo-embedded (28). In brief, tissues were incubated with an ascending gradient of sucrose and transferred to OCT compound (Tissue plus, Scigen) (10% sucrose, 30 min; 15% sucrose, overnight; 1 + 1%–15% sucrose + OCT compound, overnight; 100% OCT compound, overnight). Specimens were transferred to peel-away molds (Polysciences, Inc., Warrington, PA, USA), and frozen with ethanol–dry ice slurry. Bones were cut in a cryomicrotome (Leica CM3050, Leica, Germany) with 10 µm of thickness and transferred to superfrost slides (R. Langenbrinck GmbH, Labortechnik Medizintechnik, Emmendingen, Germany). Primary antibodies are shown in Table 1. The secondary antibodies used were biotinylated anti-mouse (1:400, Jackson Immunoresearch, Ely, UK) and rabbit anti-goat E0466 (1:400, Agilent Dako, Carpinteria, CA, USA). In both cases, heat-induced antigen retrieval in EDTA buffer (CC1 pretreatment, Roche® Ventana® Discovery products) was applied in order to uncover the different antigens and facilitate the binding of the primary antibody. Specificity of immunostaining was tested by exchanging the primary antibodies with reaction buffer or substituting them with isotype-matching immunoglobulins. Immunostainings were performed with a fully automated system (Roche^®^ Ventana^®^ Discovery) and visualized through a streptavidin-biotin method with 3,3-diaminobenzidine (DAB) as a chromophore (DAB MAP KIT, Roche^®^ enzymatic detection system). Slides were dehydrated in ascending grades of isopropanol and mounted with Entellan^®^ (Merck, Germany). Images from microscopic slides were acquired with a Zeiss AxioImager A2, Axiocam 502 and ZEN 2.3 in bright-field and differential interference contrast.

## Results

**Figure 1.**
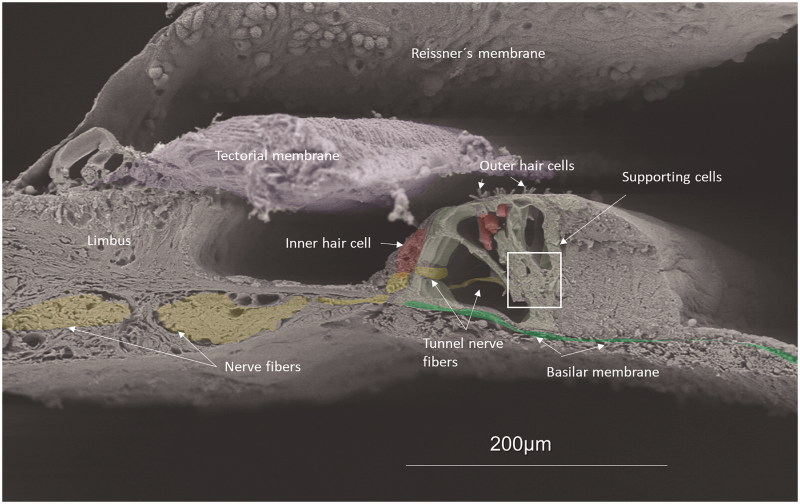
Scanning electron microscopy of the human organ of hearing (organ of Corti). Maximum resolution was to 2 nm. Different structures have been color-labeled for clarity (neurons, yellow; hair cell, red; basilar membrane, green; tectorial membrane, grey; pillar and Deiters cells, light green). Afferent synaptic terminals reach the basal pole of the IHCs. Framed area shows the basal poles where neurons innervate the OHCs. The tunnel nerve fibers are efferents from the medial olivo-cochlear portion innervating the OHCs. Efferents via the lateral olivo-cochlear portion reach the IHC synaptic terminals. Afferent fibers to the OHCs cross the tunnel basally and cannot be seen here. (*A different version of this image was published earlier in Anatomical Record [Rask-Andersen et al. 295:1791–1811 (2012)]. Permission for reuse was granted. Permission to reuse Figure 1: License Number 4633510242533; License Date: 21 July 2019; Licensed Content Publisher: John Wiley and Sons; Licensed Content Publication: The Anatomical Record: Advances in Integrative Anatomy and Evolutionary Biology*).

Scanning electron microscopy of a human organ of Corti at the low-frequency region is shown in Figure 1. Results of the expression of various subtypes of Na/K-ATPase are shown in Table 2.

**Table II.** Expression of Na/K-ATPase subtypes.

	ATPase α1	ATPase β1	ATPase α2	ATPase β2	ATPase α3	ATPase β3
SGN-p (type I)	?	+	–	–	+	NA
SGCs	+	–	–	?	–	NA
Axons	AIS	+	–	–	+	NA
Dendrites	–	+	–	–	+	NA
Nerve endings	–	+	–	–	+	NA
ISB	?	+	–	–	+	NA
OSB	+	+	–	–	+	NA
TCF	+	+	–	–	+	NA
TBF	+	+	–	–	+	NA
SGN (type II)	+	?	–	–	+	NA

ISB: inner spiral bundle; NA: not analyzed; OSB: outer spiral bundle; SGC: satellite glial cells (axons and dendrites are SGN’s neurites; nerve endings, SGN terminal fibers, and nerve endings inside organ of Corti are unmyelinated); SGN-p: spiral ganglion type I neuron perikarya; TBF: tunnel basal fibers (afferents type II fibers); TCF: tunnel-crossing fibers (efferents).

### Confocal microscopy and SR-SIM

**Figure 2.**
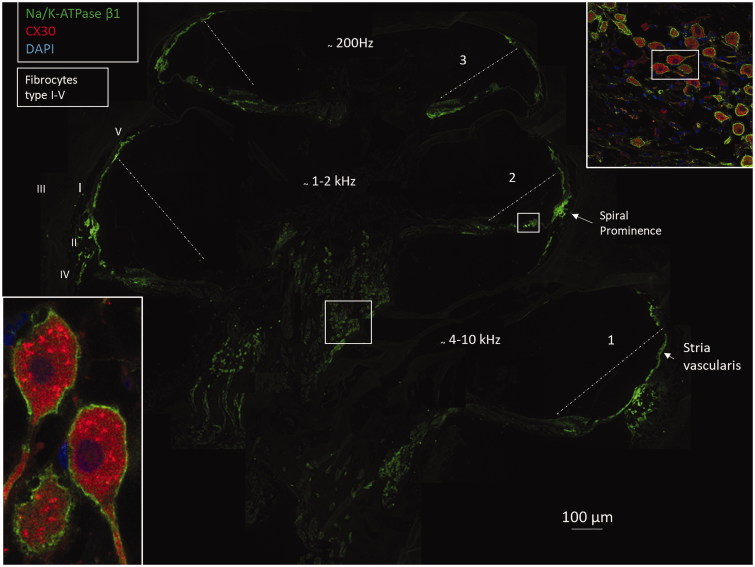
Confocal microscopy and composite micrograph showing the expression of Na/K-ATPase β1 in a mid-modiolar sections of an adult human cochlea. The interrupted line shows position of Reissner’s membrane. There is a strong expression of Na/K-ATPase at the spiral prominence, stria vascularis, and the plasma membranes of the spiral ganglion cell bodies. The ganglion cells express connexin30 (insets). Framed area in upper inset is shown in higher magnification in lower left inset. Cochlear turns are denoted by 1–3. Fibrocyte types in the lateral wall are marked I–V. Framed areas are magnified in Figure 5(C) and Supplementary Figure 2C (available online).

**Figure 3.**
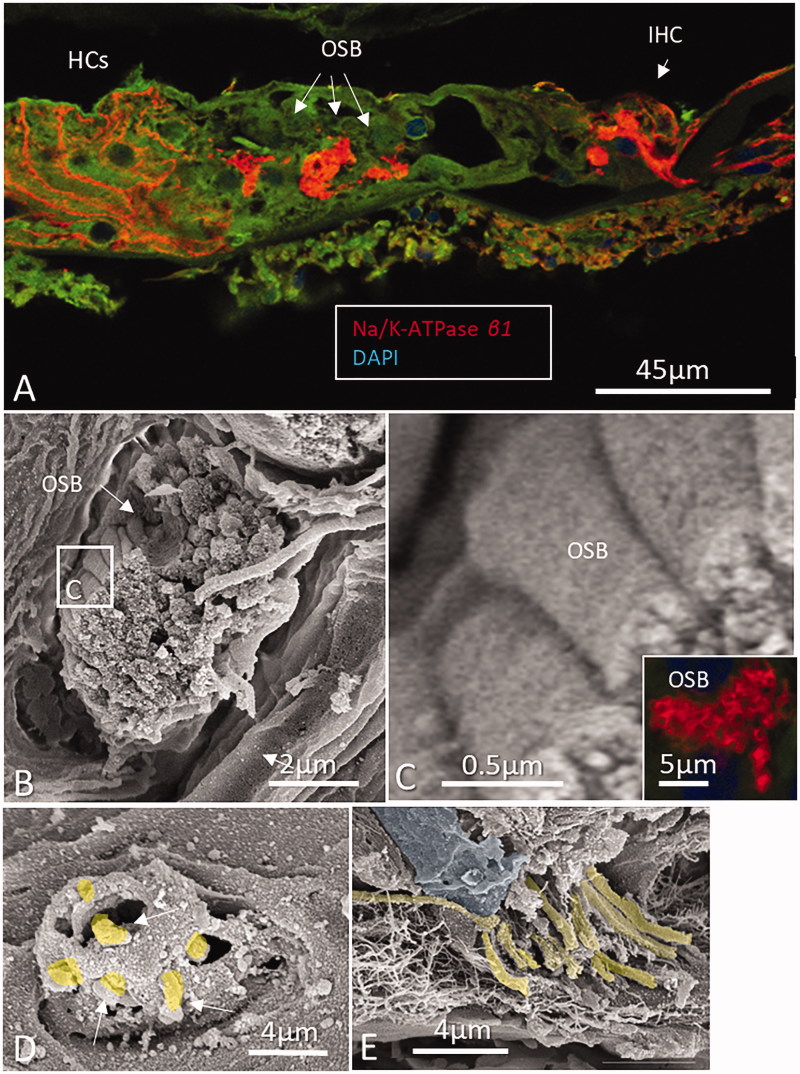
A: Confocal microscopy of Na/K-ATPase β1 expression in neurons of the human organ of Corti. The basolateral membranes of Hensen cells (HCs) are positive as well. The Corti tunnel is partly collapsed. Neurons beneath the IHC, outer spiral bundle (OSB), and tunnel spiral bundle show intense expression of the β1 isoform. B: Scanning electron microscopy of an OSB beneath the OHC. Framed area is magnified in C. Inset in D shows β1 expression of the axons. The bundle is believed to contain both efferents and afferents. D and E: Scanning electron microscopy of pre-terminal fibers (yellow) at the habenula perforata. Fiber diameter is between 0.5 and 1.0 μm.

**Figure 4.**
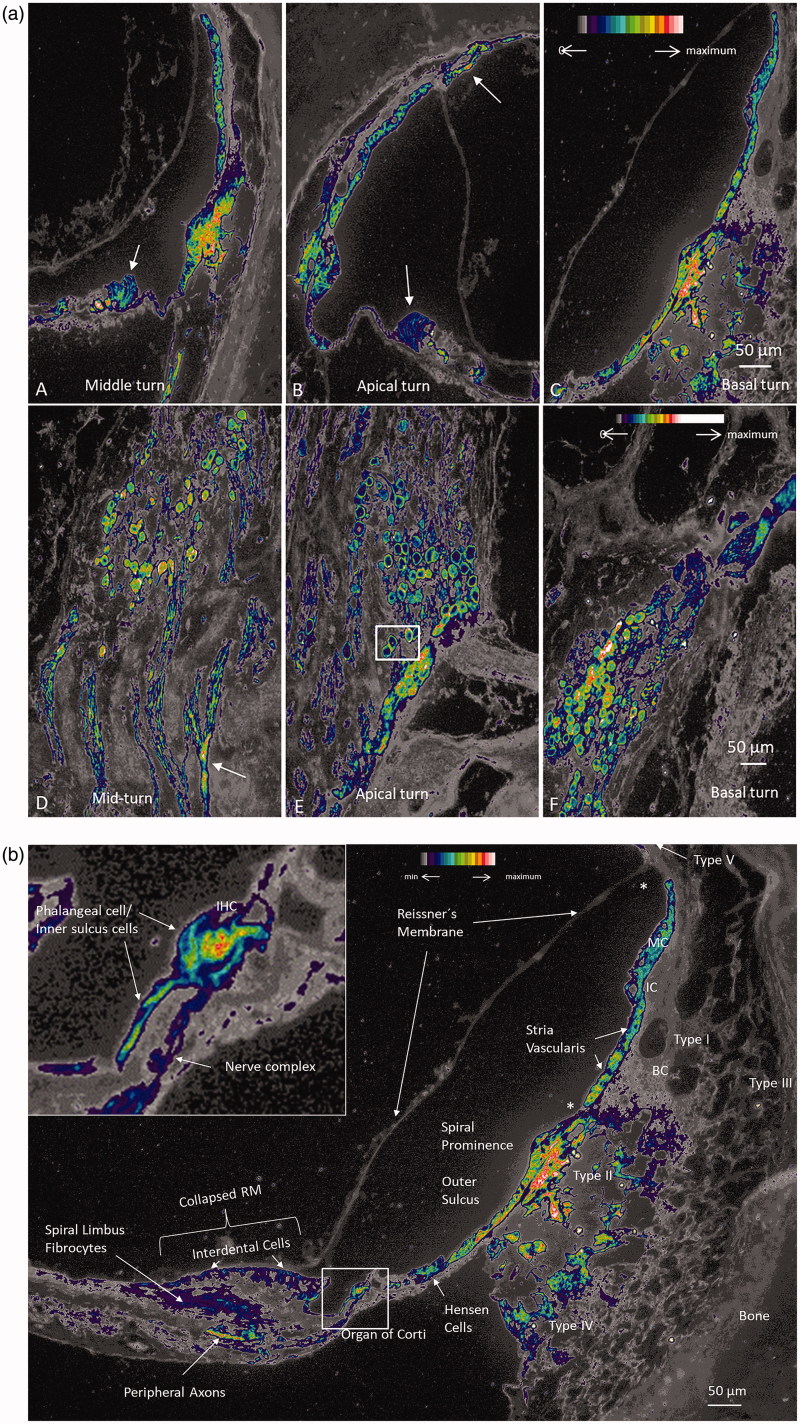
(a) A–C: Na/K-ATPase β1 expression in different turns of the human cochlea. Intensity differences are represented by false color display. There is highest intensity in type II fibrocytes beneath the spiral prominence epithelium. Hensen cells are positive (arrow in A), There is no expression of Na/K-ATPase in the Reissner’s membrane. D–F: Na/K-ATPase β1 expression in the spiral ganglion neurons at different turns showing some variation. Some cells are dislodged basally (arrow in D). Framed area in E is magnified in Figure 5(E). (b) Higher magnification of Na/K-ATPase β1 expression that was revealed in Figure 4(C). Framed area is magnified in the inset. Note lack of β1 expression in Reissner’s membrane, intermediate cells (IC), basal cells (BCs), and types I and III fibrocytes. Upper and lower limits of the stria vascularis (marked with asterisks) also lack β1 expression. Inset shows strong Na/K-ATPase activity around the IHC representing neurons and phalangeal and inner sulcus cells. Interdental cells, spiral limbus fibrocytes, and Hensen cells show moderate intensity. There is high activity in the type II fibrocytes of the lateral wall.

**Figure 5.**
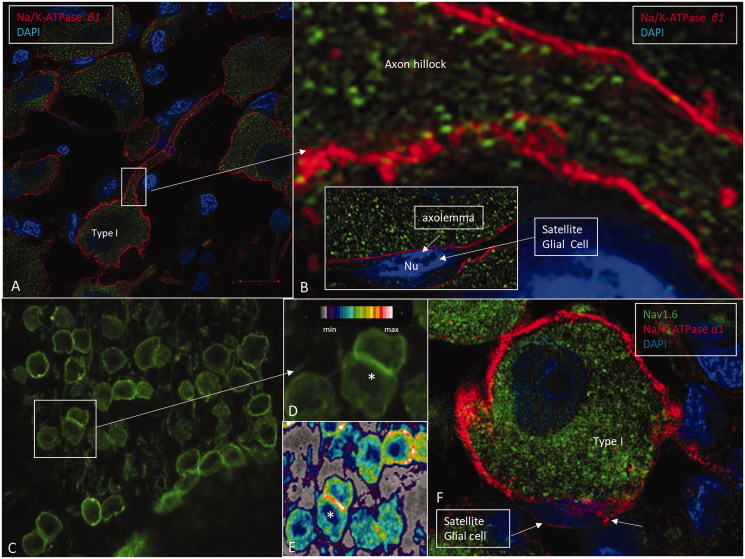
A: SR-SIM showing Na/K-ATPase β1 expression (red) in the type I cell soma plasma membranes. B: Framed area in A shows the expression of Na/K-ATPase β1 at the axon hillock in higher magnification. This isoform is not expressed in the cell membrane of the satellite glial cells (inset). C: CM of the spiral ganglion framed in Figure 2. Framed area is magnified in D. Some cells lie juxtaposed (*) and show stronger expression of Na/K-ATPase, which is also verified in the false color display in E. F: A type I spiral ganglion cell is surrounded by a satellite glial cell that shows strong expression of Na/K-ATPase α1 (red). The nerve cell body expresses Nav1.6 (green).

The mouse monoclonal Na/K-ATPase β1 antibody showed consistent, strong labeling of human cochlear tissue. A composite micrograph of a mid-modiolar section of a human cochlea demonstrated the expression and distribution of Na/K-ATPase β1 (Figure 2

). Prominent Na/K-ATPase β1 expression was noted at the spiral prominence, marginal cells of the stria vascularis, types II, IV, and V fibrocytes, spiral ganglion neurons, nerves in the organ of Corti (Figure 3), dendrites, axons, interdental cells of the spiral limbus, basolateral cell membranes of the supporting cells (Deiters and Hensen cells), Boettcher and Claudius cells, and outer and inner sulcus cells. Scanning electron microscopy showed the outer spiral bundle as well as the nerve fibers entering the organ of Corti in man (Figures 3(B–E)). β1 Expression in fibers of the outer spiral bundle was found using SR-SIM (inset of Figure 3(C)). Cross-sectioned myelinated axons co-labeled with myelin-basic protein and Na/K-ATPase β1 showed expression along the inter-nodal axoplasm including the nodes (Supplementary Figure 1, available online). No particular specializations were noted at the Ranvier nodes. There was no visible β1 expression in Reissner’s membrane, intermediate and basal cells of the stria vascularis, types I and III fibrocytes, inner and outer hair cells, pillar cells, and Deiters cells. Moreover, there was no expression at the insertion of the Reissner’s membrane at the stria vascularis and at the rim between the spiral prominence epithelium and marginal cells. The type I neuron cell bodies also expressed connexin30 (Figure 2). False color display showed the intensity of Na/K-ATPase β1 in greater detail (Figures 4 and 5(E)). The highest intensity was demonstrated at the inner hair cell–nerve junction, outer and inner spiral bundles, spiral prominence, and type II fibrocytes. There was also a high intensity in the plasma membrane of type I spiral ganglion neurons, especially at juxtaposed cell bodies (Figure 5(C–E)). SR-SIM confirmed the sub-cellular distribution of Na/K-ATPase β1 in the plasma membrane of type I cells and axoplasm (Figure 5(A–E)). No β1 isoform was expressed in satellite glial cells that otherwise showed strong expression of the α1 isoform. Alfa3 and β1 antibodies were both from mouse, so combination was not possible. Hensen cells showed strong expression of Na/K-ATPase β1 in the basolateral membranes (Supplementary Figure 2, available online). Antibodies against the voltage-gated sodium channels 1.6 (Nav1.6) labeled Ranvier nodes and the cytoplasm of the types I and II spiral ganglion neurons (Figure 5(F)).

### Light microscopy and biotin-DAB technique

#### Na/K-ATPase α3

**Figure 6.**
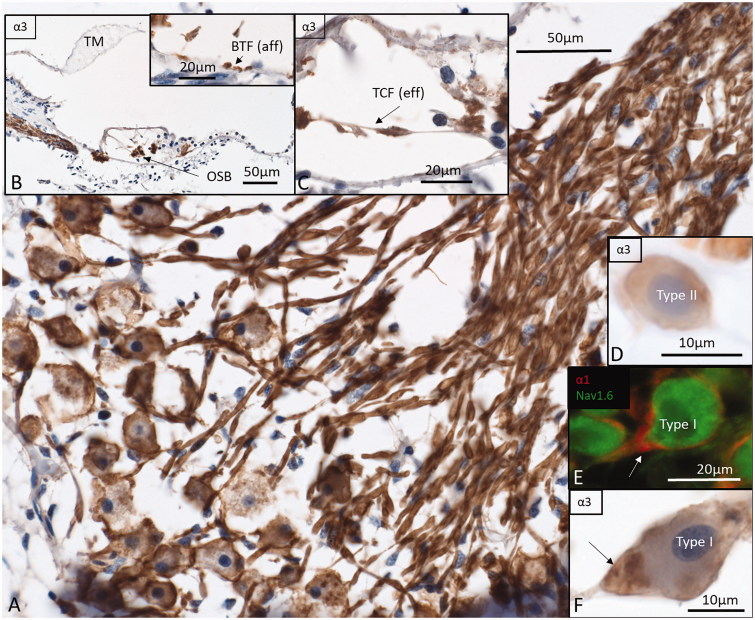
Immunohistochemistry of the human cochlea in a temporal bone obtained at autopsy. A: There is a strong expression of Na/K-ATPase α3 subunit in the spiral ganglion cell bodies, membrane, and nerve fibers. Both type I and type II cells (D) are positive. B: There is a high expression of α3 in the spiral lamina fibers and neurons beneath the IHCs and OHCs. Basal tunnel fibers (BTF-afferents) (inset in B) and efferent tunnel-crossing fibers (TCFs) (C) also express the α3 subunit. E: α1 is intensively expressed in the satellite glial cells at the axon hillock region using CM F: Corresponding type I cell shows α3 expression in the neuron at the axon hillock. OSB: outer spiral bundle; TM: tectorial membrane.

Type I neurons were positively stained around the soma as well as the peripheral and central process (Figure 6(A)). Occasionally, there was more intense immunostaining in the axon hillock region (Figure 6(F)). Type II neurons were immune-reactive confirming staining in basilar fibers (Figure 6(D)). Cross-sections in the osseous spiral lamina located α3 immunostaining in the axoplasm. In the organ of Corti, tunnel spiral bundle (TSB), outer spiral bundles (OSB) such as the basilar fibers (BF) at the base of the tunnel (type II afferents), tunnel-crossing fibers (TCF, medial efferents), and nerve fibers of the inner spiral plexus (SPI-spiral plexus) were also stained (Figure 6(B,C)). The IHCs were void of immunoreactivity, but afferent processes contacting them were highly positive.

#### Na/K-ATPase α1

IHCs, as well as nerve fibers underneath, efferent fibers in the tunnel of Corti (TCF, TSB), OSB, BFs, Hensen cells, and phalangeal cells immune-stained for α1 (Supplementary Figure 3A, available online). There was staining around the IHCs in upper middle and apical turns as well (Supplementary Figure 3A, inset). In the spiral ganglion, α1 was restricted to the region of the soma and axon initial segments (Supplementary Figure 3C). Nerve fibers were largely negative despite staining in the intra-ganglionic spiral bundle (IGSB) that is known also to contain unmyelinated efferent fibers. Some large-diameter nerve fibers in central axons contained Ranvier node-like staining with very short internode length (Supplementary Figure 3B). The median values from two different anatomy specimens showed an inter-nodal length of 7.33 µm, node length of 2.46 µm, and diameter of the fiber at the middle of the Ranvier node of 3.95 µm. Type II neurons were also positively stained for α1 and at the level of the axon hillock (Figure 6(E)).

## Discussion

To our knowledge this is the first study of electrogenic Na/K-ATPase expression in the human cochlea using SR-SIM. SR-SIM has previously been used to characterize the dynamics of Na/K-ATPase in transfected brain neurons in rat embryos (29). Due to the rarity of well-fixed human cochlear tissue, stored archival sections were used. Tissue showed excellent immunogenicity with some distortion of the organ of the Corti architecture. Glutaraldehyde gave better structural preservation but could not be used for immune analyses.

There have been several studies of Na/K-ATPase expression in the mammalian cochlea over time using various techniques (30,31). A few studies of the human cochlea have been performed (2), and the Na/K-ATPase subtypes in the human auditory periphery are largely unknown. The pattern of heterogeneity of Na/K-ATPase expression in the human auditory nerve may give additional information about its function. Weber et al. (1) performed immunostaining using rabbit polyclonal and mouse monoclonal antibodies to locate isoforms α1, α2, α3, 1, β1, and β2 using the biotin-DAB reaction. The technique detected only the β2 subunit. They found strong staining of nerve endings beneath outer hair cells (OHCs) and IHCs, satellite glial around the spiral ganglion neurons, and the basolateral membrane of stria marginal cells, and moderate reactivity along the lateral cell membrane of the Hensen cells. A strong membrane expression in Hensen cells was found in the present study. It may be relevant considering the rich number of gap junctions expressing connexin30 between these cells that may be responsible for the recirculation of K^+^ from the organ of Corti to the lateral wall (2,32).

Animal data show a modulation of Na/K-ATPase isoforms during inner ear maturation that may be related to changes in ion concentration and formation of the endo-cochlear potential (29,33). The ubiquitous α1β1 subunit combination is expressed in the basolateral membrane of the marginal cells of the stria vascularis. However, studies performed in different species suggest that stria vascularis mostly expresses β2 (1,34) or both β1 and β2 (35), while β1 is expressed in the spiral ligament. These findings were somewhat contradicted by Liu et al. (2) who found only the α1β1 subunits in the human stria. The different results may be explained by the species used and the techniques used to detect the enzyme pump. False color display shows a remarkable diversity in Na/K-ATPase intensity in various domains. Reissner’s membrane (36), basal cells and intermediate cells of the stria vascularis, types I and III spiral ligament fibrocytes, inner pillar cells, outer pillar cells, and Deiters cells lacked visible Na/K-ATPase β1 expression. Reissner’s membrane separates large ion concentrations between the endo- and perilymph. It suggests that alternate instruments exist to avoid ionic equilibration, though some α1 activity could be recognized. The high intensity of Na/K-ATPase at the spiral prominence and type II fibrocytes was striking. It is in accordance with theories of active trans-epithelial water flux and K^+^ recirculation in the lateral wall (2,37,38).

### Na/K-ATPase and cochlear nerve excitation

Na/K-ATPase plays a role in restoring ion concentration and membrane potential after the generation of action potential (12). The intensity differences found may reflect variable metabolic turnover, also noticeable in their vascular supply. The enzyme pump is estimated to consume around 50% of the total brain energy (39), depending on the rate of action potentials (40). The high-intensity display of Na/K-ATPase in the surface membrane of human spiral ganglion neurons in plasma cell membranes of juxtaposed type I cells is intriguing. Interactive transmission pathways through symmetrical and asymmetrical membrane specializations, measuring up to 3 × 2 μm in size, were described between spiral ganglion cell soma (41,42). These were frequent in the apical region and were postulated to sharpen electric signals possibly related to speech coding. In the present study, it could not be determined if the cell membrane also expressed Nav1.6. Spike generators in spiral ganglion cell bodies could compensate for the lack of myelin that should reduce signal speed across the spiral ganglion. The generators could also be activated by electric stimulation from auditory implants.

### Na/K-ATPase in satellite glial cells

In the brain, astrocytes control osmotic equilibrium by modulating the activity of Na/K-ATPase via Ca^2+^ signaling (43) and chloride shift (44). Human spiral ganglion cell bodies are surrounded by a communicating glial syncytium (45), which may act similarly to maintain ion gradients across the plasma membrane after depolarization and neuron excitation. Our results suggest that the principal heterodimer in the human auditory nerve is α3β1, while in the satellite glial cells it is α1. The β isoform could not be unequivocally established, but it is assumed to be β2 with no β1 expression in the satellite glial cells; β2 is believed to be a homologue of the adhesion molecule on glia (AMOG) (46) and is said to be the principal β isoform in Schwann cells containing either α1β2 or α2β2 heterodimers (47). Watts et al. (48) found transcripts encoding the β1 subunit in neurons, while β2 subunit mRNA expression was characteristic of glia. Alfa2 subunit mRNA was typical for glia. A transcript of α3 subunit was found in neurons, while the α1 gene was present in all cell types in the rat brain (48). Their results suggest that that there may be more than one α subunit expressed within a single cell type.

### Organ of Corti and nerve excitation

McLean et al. (49) studied the distribution of the Na/K-ATPase α subunit in rat spiral ganglion and organ of Corti. As in other neural tissues, α3β1 oligomer was expressed in inner ear neural cells. They also found the α3 isoform expressed within the membranes of the spiral ganglion cell soma, afferent terminals at the IHCs, and efferent neurons innervating the OHCs. There was an impressive Na/K-ATPase activity in the unmyelinated fibers beneath the IHCs, where action potential is believed to be generated and where voltage-gated ion channels responsible for spike generation exit the organ of Corti (50). Acoustic fibers were labeled all the way from IHC synapses and hemi-nodes to the spiral ganglion with Nav1 channels located at a short area believed to represent the spike generation immediately central to the habenula perforata. McLean et al. (49) reported similar findings in the rat, while in mice Hossain et al. (51) found Nav1.6 on unmyelinated fibers in the organ of Corti as well. They concluded that the channels generate action potentials at several locations such as the afferent endings, ganglion initial segments and nodes of Ranvier. McLean et al. (49) also demonstrated the α1 isoform in supporting cells around the IHCs together with glutamate/aspartate transporter (GLAST). Interestingly, double-labeling with a pan anti-voltage-gated sodium channel showed that α3 expression was located more peripheral and did not overlap with sodium channels at the hemi-node. This suggests that both α1 and α3 may regulate afferent-IHC synapses including GLAST. In the present study, a similar strong β1 and α1 expression was found in the nerve fiber bundles beneath the IHCs and OHCs (inner and outer spiral bundles). Nakazawa et al. (52) used electron microscopic immunogold cytochemistry in gerbils and found Na/K-ATPase detectable in the neurilemma of both myelinated and unmyelinated afferents but not in efferent nerve processes beneath hair cells. We found strong Na/K-ATPase expression in all efferent systems, including the small unmyelinated and myelinated fibers in the intra-ganglionic spiral bundle in the Rosenthal canal. Moreover, we found expression of α3 and β1 isoforms in type II cells and OHC afferents, although we did not perform co-staining with anti-peripherin antibodies (53). The co-expression of α1 and α3 among organ of Corti neurons may reflect particular demands and characteristics of these neurons. The neurites are only partly embedded in supporting cells that are thought to take over glial cell function. Therefore, excitation, spike propagation, and membrane potential recovery at these projections must be rather unique. Previous studies have shown a co-localization with AMPA receptors in neurons, where Na^+^ clearance is needed after depolarization (54). A fine-tuning of the responses can explain the co-expression of α1 and α3 in afferent and efferent fibers at the level of the organ of Corti. Furthermore, IHC activity may require a high expression level of α1 subunit in phalangeal cells with high affinity for K^+^ for efficient ion recirculation to maintain micro-homeostasis. Neuronal α3 appears to be optimized for high-intensity neuronal firing supporting active efflux of Na^+^ when high concentrations are present after the generation of action potentials. The pore forming α1 subunit was described to have higher affinity for K^+^, while α3 has lower Na^+^ affinity (55). Different Na/K-ATPase isoform combinations could more precisely shape the response to a variety of complex stimuli affecting the ear.

**Figure 7.**
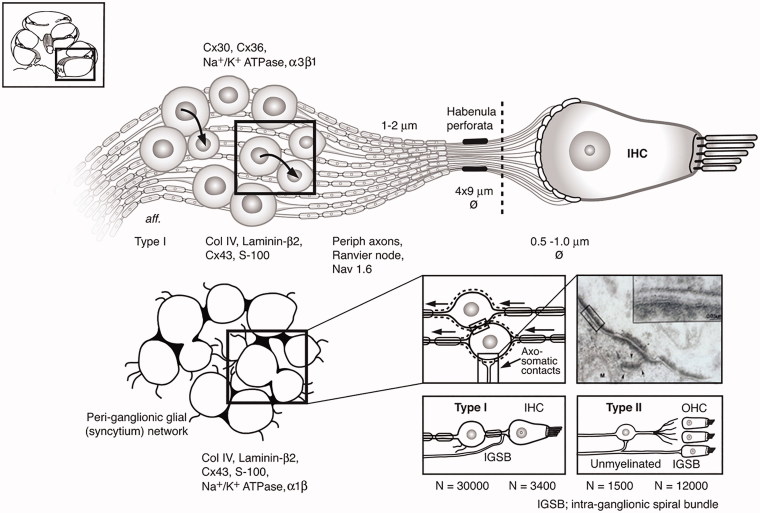
Molecular expression and organization of the principal afferents and ganglion satellite glial syncytium in the human cochlea. Each IHC is innervated by approximately 10–15 primary afferents (large or type I SGNs) with 15–18 synaptic terminals. Small or type II neurons converge from 12,500 OHCs. Unlike in most vertebrates, the human SGN cell bodies are surrounded by thin SGCs forming a more or less continuous network. Axo-somatic synaptic contacts exist on both types I and II cell bodies. Areas with no separating glia layer between type I cell bodies exist in man with both symmetric and asymmetric soma-somatic membrane (41, 42). These connections may couple individual cells electrically. Transmission electron microscopy inset shows axo-somatic contact with synapse-like membrane specializations (*with permission from Auris Nasus Larynx. Rask-Andersen, et al. 1997;24:1–11*). The intra-ganglionic spiral bundle (IGSB) contains efferent fibers from the olivo-cochlear bundle. They leave the inferior vestibular nerve in the internal acoustic canal to reach the cochlear nerve. (*Permission to reuse TEM inset in Figure 7: License Number: 4633560923178; License Date: 21 July 2019; Licensed Content Publisher: Elsevier; Licensed Content Publication: Auris Nasus Larynx; Licensed Content Title: Nerve fibre interaction with large ganglion cells in the human spiral ganglion. A TEM study*).

From the present investigation and previous data (Figure 7), one may assume that both organ of Corti neurons and spiral ganglion may act as excitation sites of extra-cellular electric stimulation with auditory implants. Further analyses are underway to better understand the location and distribution of spike generators in the human cochlea.

## Supplementary Material

Supplemental Material

Supplemental Material
