# Supplementary figures and images for: Expression of Na/K-ATPase subunits in the human cochlea: a confocal and super-resolution microscopy study with special reference to auditory nerve excitation and cochlear implantation
Source: Ups J Med Sci. 2019 Aug 28;124(3):168–79. doi: 10.1080/03009734.2019.1653408 (PMC6758701; doi:10.1080/03009734.2019.1653408)

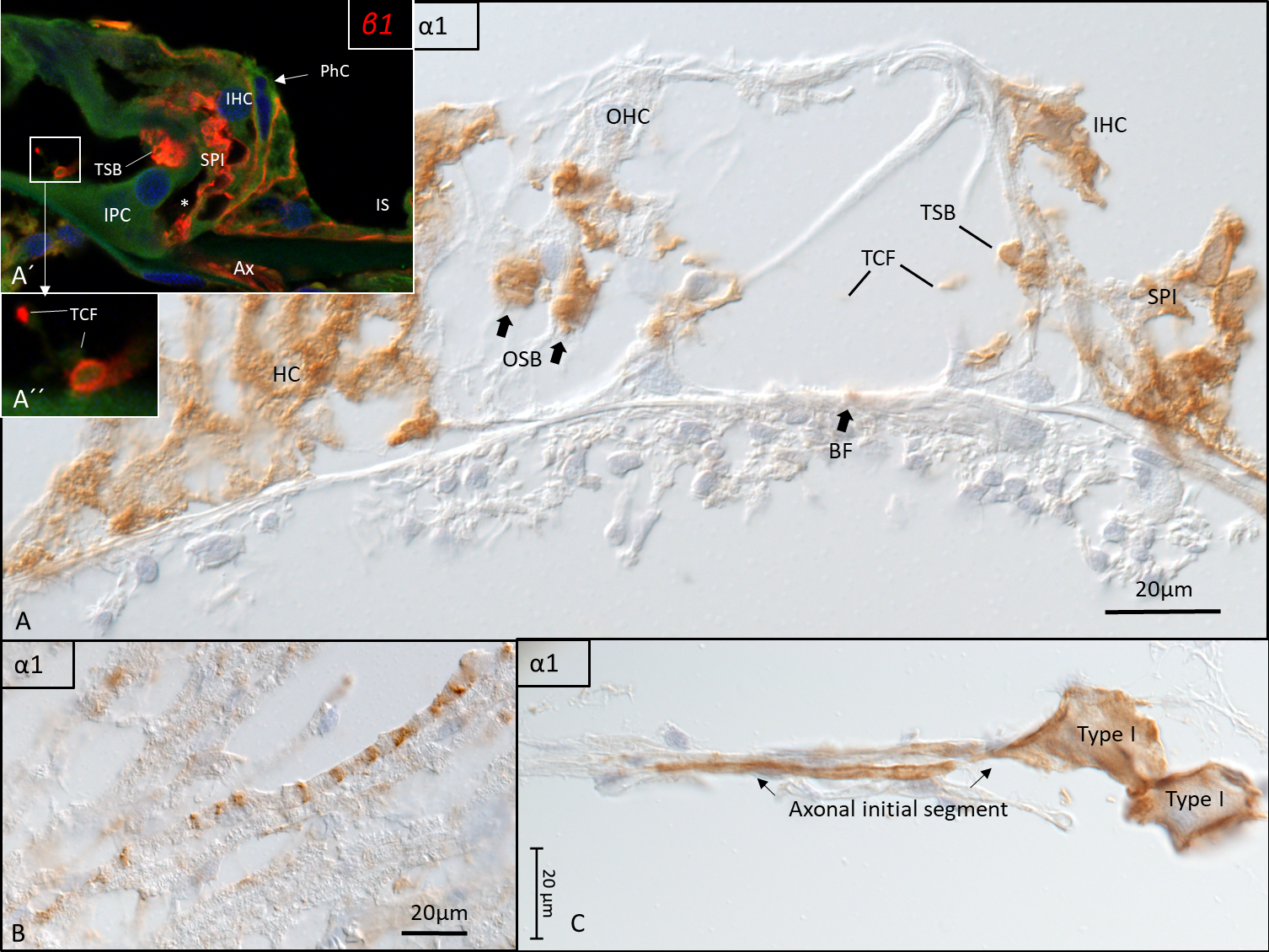

Supplement: Supplemental Material [file IUPS_A_1653408_SM8055.zip › Supplementary Fig 3.tiff]

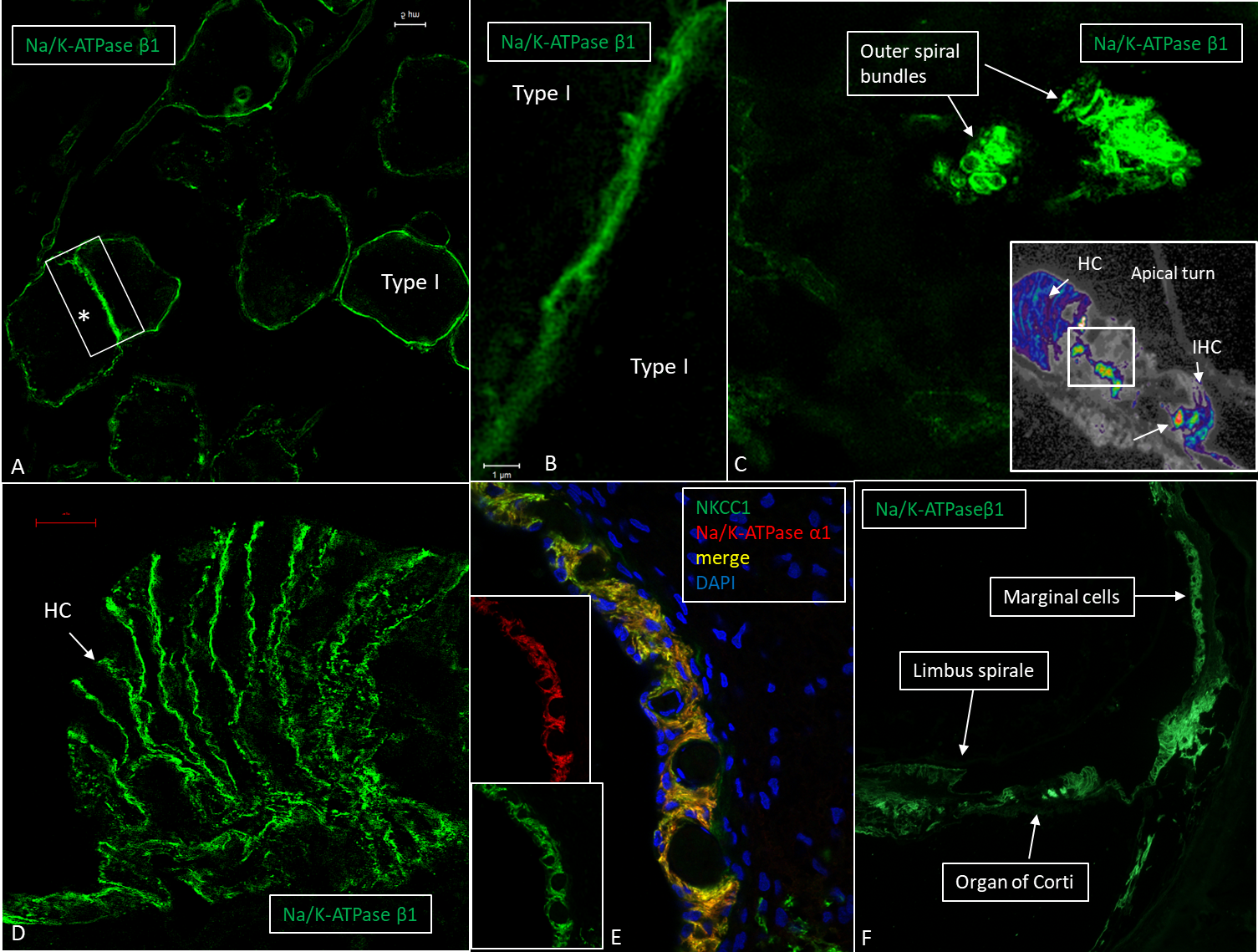

Supplement: Supplemental Material [file IUPS_A_1653408_SM8055.zip › Supplementary Fig 2.tiff]

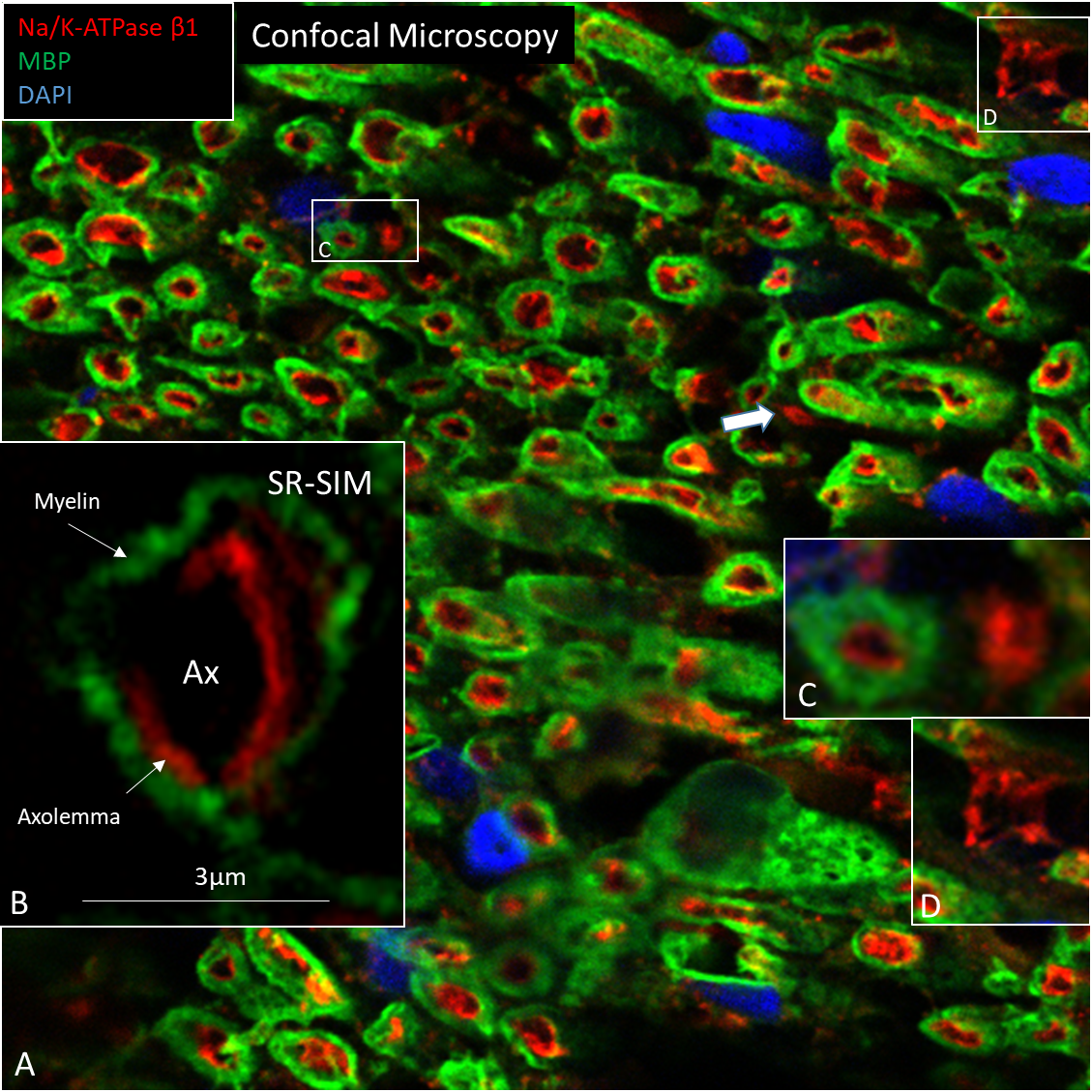

Supplement: Supplemental Material [file IUPS_A_1653408_SM8055.zip › Supplementary Fig 1.tiff]
